# Clinicopathologic and Treatment Features of Long-Term Surviving Brain Metastasis Patients

**DOI:** 10.3390/curroncol28010054

**Published:** 2021-01-18

**Authors:** Archya Dasgupta, Jayson Co, Jeff Winter, Barbara-Ann Millar, Normand Laperriere, Derek S. Tsang, Monique van Prooijen, Andrei Damyanovich, Robert Heaton, Catherine Coolens, Mark Bernstein, Paul Kongkham, Gelareh Zadeh, Alejandro Berlin, Tatiana Conrad, Fabio Y. Moraes, David B. Shultz

**Affiliations:** 1 Department of Radiation Oncology, University of Toronto, Toronto, ON M5G2M9, Canada; archya1010@gmail.com (A.D.); jayson.co@yahoo.com (J.C.); jeff.winter@rmp.uhn.ca (J.W.); Barbara-Ann.Millar@rmp.uhn.ca (B.-A.M.); Norm.Laperriere@rmp.uhn.ca (N.L.); derek.tsang@rmp.uhn.ca (D.S.T.); Andrei.Damyanovich@rmp.uhn.ca (A.D.); Robert.Heaton@rmp.uhn.ca (R.H.); catherine.coolens@rmp.uhn.ca (C.C.); alejandro.berlin@rmp.uhn.ca (A.B.); Tatiana.Conrad@rmp.uhn.ca (T.C.); fabio.ynoedemoraes@kingstonhsc.ca (F.Y.M.); 2 Radiation Medicine Program—Princess Margaret Cancer Centre, University Health Network, Toronto, ON M5G2M9, Canada; Monique.vanProoijen@rmp.uhn.ca; 3 Department of Medical Biophysics, University of Toronto, ON M5G2M9, Canada; 4 Division of Neurosurgery—Toronto Western Hospital, University of Toronto, Toronto, ON M5G2M9, Canada; Mark.Bernstein@uhn.ca (M.B.); Paul.Kongkham@uhn.ca (P.K.); Gelareh.Zadeh@uhn.ca (G.Z.)

**Keywords:** brain metastasis, long-term survivors, molecular characteristics, systemic therapy, targeted therapy, immunotherapy

## Abstract

Background: The purpose of our study was to characterize clinical features among brain metastasis (BM) patients who were long term survivors (LTS). Methods: We reviewed a registry of BM patients referred to our multidisciplinary BM clinic between 2006 and 2014 and identified 97 who lived ≥ 3 years following BM diagnosis. The clinical and treatment characteristics were obtained from a prospectively maintained database, and additional information was obtained through review of electronic medical records and radiologic images. Survival analyses were performed using the Kaplan-Meier method. Results: Median follow up for LTS was 67 months (range 36–181). Median age was 54 years, 65% had single BM, 39% had stable extracranial disease at the time of BM treatment, and brain was the first site of metastasis in 76%. Targetable mutations were present in 39% of patients and 66% received treatment with targeted-, hormonal-, or immuno-therapy. Brain surgery at the time of diagnosis was performed in 40% and stereotactic radiosurgery (SRS) or whole brain radiotherapy (alone or combination) in 52% and 56%, respectively. Following initial BM treatment, 5-year intracranial disease-free survival was 39%, and the cumulative incidence of symptomatic radio-necrosis was 16%. Five and ten-year overall survival was 72% and 26%, respectively. Conclusion: Most LTS were younger than 60 years old and had a single BM. Many received treatment with surgery or targeted, immune, or hormonal therapy.

## 1. Introduction

Brain metastases (BM) affect approximately 30% of cancer patients and are especially common in lung, breast, kidney, gastrointestinal cancers, and melanoma [1,2]. The incidence of BM is increasing, due in part to improved neuroimaging and longer overall survival among cancer patients due to improved systemic therapies [3]. Historically, whole-brain radiotherapy (WBRT) was the principal management for all but single BM, irrespective of disease burden [4]. Today, stereotactic radiosurgery (SRS) for oligometastatic BM is the standard of care, in part because it results in lower rates of neuro-cognitive toxicity compared to WBRT and improved local control, albeit with worse distant brain control [5,6,7]. Furthermore, newer systemic therapies with improved central nervous system activity are increasingly used in the management of BM [8,9,10].

Few published reports have described the characteristics of BM patients who were long-term survivors (LTS) [11,12,13,14,15,16,17]. The purpose of our study was to characterize clinical features among BM patients who were LTS patients treated within our institution.

## 2. Methods and Materials

### 2.1. Patient Selection

With institutional ethics committee approval (University Health Network, protocol number 18-5741.1), we identified 97 LTS (≥3 years) from a prospectively maintained registry of patients treated for BM at Princess Margaret Cancer Center between January 2006 and December 2014. This registry includes all patients referred to our multidisciplinary BM clinic, which focuses on patients eligible for SRS. All patient, disease, and treatment-related information was retrieved from the database and, when required, verified through the evaluation of electronic medical records and radiologic images.

### 2.2. Treatment and Follow-up Protocols

Given the prolonged study period, management algorithms evolved over time. Treatment decisions were guided by multi-disciplinary discussions involving radiation oncologists, neurosurgeons, and medical oncologists. Our current treatment protocols for BM prescribe stereotactic radiosurgery (SRS) for limited numbers of metastases (<10) in patients with good performance status (PS). Many patients in our total cohort (56%) treated during an earlier era received WBRT alone or in combination with SRS or surgery. Likewise, some patients were diagnosed during an era that preceded molecular profiling and the availability of targeted agents or immunotherapy. Surgery was generally recommended for patients with larger or symptomatic metastases. Following treatment completion, patients were followed clinically and radiologically with magnetic resonance imaging (MRI) brain or contrast-enhanced computed tomography (CT) every 3 months for the first 2 years and, if stable, every 6–12 months subsequently. During follow up, all patients were reviewed by a multi-disciplinary team to determine disease control status and toxicity, and to formulate salvage treatment strategies when required. 

### 2.3. Statistical Analysis

Analyses were performed using the Statistical Package for the Social Sciences (SPSS V21). For survival analysis, the Kaplan Meier product-limit method was used, starting with the date of the radiological diagnosis of BM. Log-rank test and Cox regression tests were used for univariate and multivariate analyses, respectively, to find features with a significant impact on survival. A two-sided “*p*-value” of <0.05 was considered to be statistically significant.

We designated extracranial disease or primary disease as “controlled” if there was no measurable disease progression 3 months prior to or following BM diagnosis. When the interval between diagnosis of primary malignancy and BM was ≤3 months, we classified BM as having been diagnosed at disease presentation. The administration of systemic therapy with respect to BM management was divided into three categories: before (>3 months prior to diagnosis), concurrently (+/− 3 months from diagnosis of BM), or later (>3 months).

## 3. Results

### 3.1. Survival

**Figure 1:**
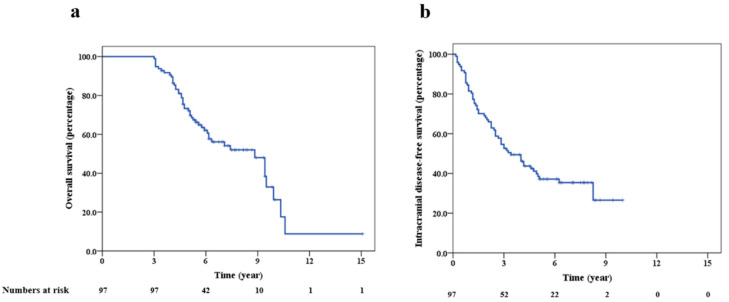
Kaplan Meier survival plots showing overall survival (**a**) and intracranial disease-free survival (**b**) for long-term survivors.

Among the 97 patients identified within our prospective database who survived greater than 3 years following their diagnosis of BM and were thus identified as LTS, median follow up was 67 months (range 36–181 months). Five-year and ten-year overall survival (OS) in the LTS cohort was 72% and 26%, respectively (Figure 1a), and median survival was 106 months (95% confidence interval, 79–133 months). At the time of last analysis, 42 patients were alive without active disease (intracranial or extracranial), eight were alive with progressive disease (in two cases intracranial), and 47 had died. Of patients who died, 25 had an MRI brain within three months of their deaths, which in 16 (64%), showed no active or progressive intracranial disease. We also investigated whether OS among LTS correlated with the era during which patients initially presented and found no difference (Supplementary Figure S1).

### 3.2. Patient Clinical and Treatment Features

Demographic, disease, and treatment characteristics of LTS are summarized in Table 1. Median age was 54 years. The performance status at the time of BM diagnosis was eastern cooperative oncology group (ECOG) 0 or 1 in 75% of patients. The median number of BM at diagnosis was 1 (range 1 to 12). The most common primary cancers were lung (52%), breast (25%), thyroid (7%), and cutaneous melanoma (5%). At BM diagnosis, in 39% of patients, there was no evidence of active extracranial disease. In 76% of patients, BM was present at the time when metastatic disease was first diagnosed. 

Targetable mutations and hormonal receptor positivity (among breast cancer patients) were seen in 39% and 67%, respectively. Table 2 describes the frequency of targetable mutations among non-small cell lung cancer, breast cancer, and melanomas. Epidermal growth factor receptor (EGFR) mutation and anaplastic lymphoma kinase (ALK) rearrangement were seen in 41% and 8% of patients with lung cancer, respectively. Among breast cancer patients, hormone receptor positive (HR)+/human epidermal growth factor receptor 2 (Her 2)+, HR+/Her2-, HR-/Her2+, and triple negative subtype was present in 25%, 42%, 25%, and 8%, respectively. Targeted therapy, hormonal therapy, or immunotherapy was administered to 64 (66%) of patients (alone or combined with chemotherapy in 16 and 48 patients, respectively) at some time-point following their cancer diagnosis. Of patients receiving targeted, immuno-, or hormonal therapy, treatments were initiated greater than 3 months before the diagnosis of BM, at the time of BM diagnosis, or 3 months after BM diagnosis in 41%, 47%, and 12% of patients, respectively. The details of systemic therapy administered at some point to LTS patients are described in Supplementary Table S1. Initial BM treatments are summarized in Table 3. Forty percent of patients underwent surgical resection of their BM at diagnosis, and SRS and WBRT was administered to 52% and 56%, respectively (alone or in combination). Seven patients did not receive any form of brain radiation; five patients were treated with systemic therapy alone, and two underwent surgery alone.

### 3.3. Intracranial Outcomes 

Median radiologic follow up was 65 months (range 14–168 months). Intracranial disease-free survival (iDFS) at 3 and 5 years was 53% and 39%, respectively (Figure 1b). Sixty-two patients (64%) had radiological evidence of intracranial progression following their initial treatment; median time to recurrence was 24 months (range 2–99 months). Forty-four percent of patients had a single episode of intracranial failure following their initial treatment; the median number of failures was two (range 1–6). As the time of intracranial progression following their initial BM treatment, SRS, WBRT, surgery, and partial brain radiation was administered to 46, 18, 17, and 9 patients, respectively (alone or in combination). The five-year OS for patients who experienced intracranial progression after their first treatment for BM was 68%, compared to 80% for patients who did not (*p* = 0.22). 

### 3.4. Long-Term Toxicity 

Fifteen patients developed symptomatic radio-necrosis (RN). The 2-year and 5-year cumulative incidences of RN was 10% and 16%, respectively. Nine patients had RN after the first course of treatment (two received SRS alone and seven had a combination of surgery/SRS with WBRT) and the remaining six developed RN following subsequent salvage treatments. Nine patients with RN were treated with steroids alone and six required surgery; two out of 15 patients with RN suffered worsening of pre-existing neurologic deficits during follow up. One patient developed severe dementia following a second course of WBRT. 

## 4. Discussion

Brain metastases are generally associated with a dismal prognosis [2,3], but some patients have prolonged survival. Several prognostic indices have been developed to stratify patients and predict outcomes, including the graded prognostic assessment (GPA) [18,19,20]. The best surviving groups in recently reported disease-specific GPAs for lung adenocarcinoma, breast adenocarcinoma, and melanoma patients had median OS estimates of 47, 25, and 34 months, respectively [21,22,23]. These updated GPA classifications estimate markedly improved OS compared to the preceding models and incorporate molecular classifications. Generally speaking, specific molecular subtypes susceptible to targeted agents have the best outcomes.

Several previous reports have described patients who survived greater than two–three years following BM diagnosis11–17 (Table 4). In most studies, rates of OS among LTS were relatively stable during the three to five years following BM diagnosis. Furthermore, Kotecha et al. reported that 41% of patients who survived at least five years were alive at 10 years [17]. In our study, 42 patients were alive and without active disease at their last follow up. 

Intracranial disease burden is an important prognostic factor for BM patients [11,12,13,17]. In our cohort, 65% of LTS had a single metastasis. Kotecha et al. reported that 87% of 10-year survivors had a single BM at diagnosis, compared to 42% who survived less than 5 years [17]. These results confirm the relevance of this variable in prognostic indices like the GPA and score index for radiosurgery (SIR) [24,25]. 

The impact of treatment modality on survival for LTS is uncertain. Hall et al. reported that surgery and WBRT were associated with LTS [11]. In a series reported by Enders et al., the frequency of LTS among surgical patients was also relatively high (18%) [16]. Likewise, in our series, 40% of patients underwent surgery. This result, however, may be affected by selection bias insofar as the determination of surgical candidacy reflects pre-surgical performance status. 

Kotecha et al. noted that SRS has a positive impact on survival from BM compared to WBRT. In our series, 50% of LTS underwent treatment with SRS. However, treatment with WBRT did not correlate with decreased rates of survival. Investigating the role of WBRT in LTS is important, given its potential to cause cognitive toxicity. In general, for patients with a limited number of BM, SRS has become the preferred modality because it results in improved neurocognitive outcomes compared to WBRT [5,6,7,26]. In particular, SRS may be more appropriate for patients predicted to be LTS, given that the potential for late toxicities from WBRT. 

Finally, we report the molecular characteristics and use of specific systemic therapy (targeted, hormonal, or immunotherapy) in our LTS cohort. In addition to the well-documented activity of tyrosine kinase inhibitors against BM [27], a recent phase 2 study with 94 patients of melanoma demonstrated >50% response in nonirradiated BM treated with nivolumab and ipilimumab. In a meta-analysis of 1132 patients with melanoma BM, Rulli et al. reported that combination immunotherapy resulted in improved survival compared to mono-immunotherapy and combination targeted therapies [28]. In our study, among 89 LTS with known molecular features, 54% had a drug-targetable mutation or hormone receptor positivity and 66% received targeted, hormonal, or immunomodulating therapies. This may reflect, in part, the CNS activity of some targeted agents. The details of individual systemic agents and the timing of therapy in relation to the diagnosis of BM have been described in Supplementary Table S1. The most commonly used targeted agents included gefitinib and erlotinib in lung cancer and trastuzumab in breast cancer. Tamoxifen was the most commonly used hormonal agent. Given the small number of patients with primary cancers containing specific molecular features, we did not examine the effect of specific systemic agents on survival. 

### Strength and Limitations

Our study represents one of the largest and most well-characterized exclusively LTS cohorts. However, the patients included in this analysis have different cancers with diverse molecular features, which makes all but general conclusions difficult. Our study would be strengthened by the inclusion of a control group of patients from our database with median survival. With that we would have had the ability to compare that group’s clinical and treatment-related characteristics with that of the LTS cohort. Without the inclusion of a control group, it is difficult to conclude the characteristics that are specifically associated with LTS. Furthermore, a dedicated evaluation of long-term neuro-cognitive complications in LTS, functionality, occupational rehabilitation and the impact of different treatment modalities, would have been informative. In addition, patients included in this registry were referred to our BM clinic and as such had a high proportion of patients who were considered to have a favorable prognosis, including patients with a single brain metastasis. 

## 5. Conclusions

The majority of long-term BM survivors in our cohort were younger than 60 years of age and had a single BM at diagnosis. Many patients were treated with surgical resection or targeted, hormonal, or immune modulating therapies. LTS represent a distinct subset of BM patients for whom treatment is more than palliative and for whom the long-term side effects and risks of treatment should be carefully considered. 

## Figures and Tables

**Table 1:** Details of various patient, disease, and treatment characteristics in the long-term survivors.

Feature	Long-Term Survivors (*n* = 97)
Age	
<60 years	69 (71%)
60 years or more	28 (29%)
Gender	
Male	23 (24%)
Female	74 (76%)
ECOG (during diagnosis of BM)	
0–1	73 (75%)
2 or more	13 (14%)
Unknown	11 (11%)
Number of intracranial lesions (at time of BM diagnosis)	
Single	63 (65%)
Multiple	34 (35%)
Primary	
Lung	51 (52%)
Breast	24 (25%)
Thyroid	6 (7%)
Melanoma (skin)	5 (5%)
Kidney	3 (3%)
Unknown primary	3 (3%)
Gynecological malignancies	2 (2%)
Male genitourinary	1 (1%)
Gastrointestinal	1 (1%)
Head neck	1 (1%)
Extracranial disease status (at the time of BM diagnosis)	
Controlled	38 (39%)
Uncontrolled	59 (61%)
Interval from cancer diagnosis to detection of BM	
Median (range)	15 (0–266) months
First site of metastasis	
Brain	74 (76%)
Others	23 (24%)
Molecular characteristics	
Targetable mutations	
Yes	38 (39%)
No	51 (53%)
Unknown	8 (8%)
Hormonal receptors (among patients with breast cancer)	
Yes	16 (67%)
No	8 (33%)
Unknown	8 (8%)
Systemic therapy ^a^	
Targeted therapy/Hormonal therapy/Immune therapy	64 (66%)
None of the above/Unknown	33 (34%)
Stereotactic radiosurgery ^a^	
Yes	50 (52%)
No	47 (48%)
Whole brain radiotherapy ^a^	
Yes	54 (56%)
No	43 (44%)
Surgery ^a^	
Yes	39 (40%)
No	58 (60%)

ECOG: Eastern cooperative oncology group; BM: Brain metastasis. before, during, or after diagnosis of brain metastasis. ^a^ intracranial treatment at the time of initial diagnosis of brain metastasis.

**Table 2:** Frequency of commonly detected driver mutations between lung, breast, and melanoma primaries for the long-term survivors.

Molecular Characteristics	Long-Term Survivors
Lung primary	(*n* = 51)
EGFR mutation	21 (41%)
ALK rearrangement	4 (8%)
EGFR/ALK-	21 (41%)
Unknown	5 (10%)
Breast primary	(*n* = 24)
ER/PR +/Her 2+	6 (25%)
ER/PR+/Her 2-	10 (42%)
ER/PR-/Her 2+	6 (25%)
Triple-negative	2 (8%)
Unknown	0 (0%)
Melanoma	(*n* = 5)
BRAF+	0 (0%)
BRAF-	2 (40%)
Unknown	3 (60%)

EGFR: Epidermal growth factor receptor; ALK: Anaplastic lymphoma kinase; ER: Estrogen receptor; PR: Progesterone receptor; Her 2: Human epidermal growth factor receptor 2.

**Table 3:** Details of intracranial treatment during the initial diagnosis of brain metastases for the long-term survivors.

Intracranial Treatment	Long-Term Survivors (*n* = 97)
SRS alone	29 (30%)
Surgery alone	2 (2%)
WBRT alone	15 (15%)
Surgery with SRS	7 (7%)
Surgery with WBRT	25 (26%)
SRS and WBRT	9 (9%)
Surgery with SRS and WBRT	5 (5%)
Systemic therapy alone	5 (5%)

SRS: Stereotactic radiosurgery; WBRT: Whole brain radiotherapy.

**Table 4:** Selected studies reporting long term survivors of brain metastases.

Study	Cut-off for LTS	No of Patients	Percentage of Patients	Most Common Primary Sites	Patients Receiving WBRT	Patients Receiving SRS	Patients Undergoing Surgery	Crude rate Neurologic Death	Comments/Prognostic Factors
Hall et al. (2000) [11]	2 years	51	6.9% (2 years)	1. NSCLC 2. Breast 3. Melanoma/ renal cell carcinoma	98%	8%	57%	22%	1. Ovarian carcinoma patients had the highest rate of survival among cohort. 2. Single lesion, surgery, and WBRT were factors associated with LTS.
Lutterbach et al. (2002) [12]	2 years	2 years-48 3 years-25 5 years-12	2.8% (3 years)	1. NSCLC 2. Breast	98%	0%	73%	NA	1. For patients with single lesion, radiation boost was considered following WBRT. 2. Survival was best patients with a single brain metastasis
Kondziolka et al. (2005) [13]	4 years	44	6.5% ^a^ (4 years)	1. Lung 2. Breast 3. Kidney	86%	100%	NA ^b^	4%	Compared with a cohort of patients surviving < 3 months, LTS had better KPS, fewer metastases, and a lower extracranial disease burden.
Chao et al. (2006) [14]	5 years	32	2.5% (5 years)	1. NSCLC 2. Breast 3. Melanoma	66%	28%	69%	0% (for 10-year survivors)	1. Prognostic factors were compared to patients surviving <5 years. 2. Female gender, RPA class 1, surgery, and SRS were associated with better survival.
Niemiec et al. (2011) [15]	3 years	19	2% ^c^	NSCLC	79%	32%	53%	33%	1. Compared with control group of patients with brain metastases from lung cancer. 2. Female sex, RPA class 1, adenocarcinoma, control of primary tumour and no extracranial metastasis was associated with LTS.
Enders et al. (2016) [16]	2 years	21	18%	NSCLC	81%	0%	100%	NR	Surgery for primary tumour was the only significant factor associated with LTS.
Kotecha et al. (2016) [17]	10 years	5 years-56 10 yrs-23	1.2% (10 years)	1. NSCLC 2. Melanoma 3. Breast/unknown primary	52%	30%	70%	0% (for 10-year survivors)	Female gender, single brain metastasis and SRS were associated with better overall survival
Current study (2020)	3 years	3 years-97 5 years-64	16% (5 years)	1. NSCLC 2. Breast 3. Thyroid	56%	52%	40%	36% ^d^	71% of the LTS were <60 years, 65% had single BM at the time of diagnosis, 76% had brain as the first site of metastatic disease, 39% had targetable mutations, 66% received targeted/hormonal and immunotherapies.

LTS: Long-term survivors; WBRT: Whole brain radiotherapy; SRS: Stereotactic radiosurgery; HR: Hormonal receptor; NSCLC: Non-small cell lung carcinoma: Brain metastasis; NA: Not available; OS: Overall survival. ^a^ The cohort was chosen from a selected group of patients who had undergone SRS treatment. ^b^ the number of patients who had surgery at the time of presentation of brain metastasis is unknown. ^c^ approximate value according to the authors. ^d.^ Defined as any radiologically active CNS disease within three months before death.

## Supplementary Materials

The following are available online at https://www.mdpi.com/1718-7729/28/1/54/s1, Figure S1: Kaplan Meier survival plot showing a comparison of overall survival for patients diagnosed with brain metastasis during 2003–2010 and 2011–2014. Table S1: Systemic therapies used for long-term survivors. Table S2: Correlation of different factors with intracranial disease-free survival and overall survival for the long-term survivors.
