# Supplementary material for: Clinicopathologic and Treatment Features of Long-Term Surviving Brain Metastasis Patients
Source: Curr Oncol. 2021 Jan 18;28(1):549–59. doi: 10.3390/curroncol28010054 (PMC7903267; doi:10.3390/curroncol28010054)
Supplement: Supplementary file 1 [file curroncol-28-00054-s001.pdf]

Table S1. Systemic therapies used for long-term survivors.

**Systemic therapies**	
**Individual therapies**	**Number of patients receiving (n=84)**
**Targeted agents**	
Erlotinib	11
Gefitinib	13
Crizotinib	4
Trastuzumab	9
Sunitinib	5
Lenvatinib	3
Sorafenib	3
Osimertinib	3
Other agents	8[a]
**Immunomodulators**	
IFN	3
Durvalimumab	3
Other immunotherapy (or immunomodulator) agents	6[b]
Radioactive iodine	4
**Hormonal therapy**	
Tamoxifen	14
Letrozole	5
Exemestane	2
**Others**	
Platinum-based chemotherapy	31
Taxane-based chemotherapy	14
Cyclophosphamide based chemotherapy	21
Radioactive iodine	4
**Number of lines of systemic therapy used[c]**	
Single	22
Multiple lines	55
**Time of initiating therapy (targeted/ immunotherapy/ hormonal) (n=64)**	
Prior to diagnosis of brain metastasis	26 (41%)
At the time of brain metastasis diagnosis (+/- 3 months)	30 (47%)
After the diagnosis of brain metastasis (> 3 months)	8 (12%)

[a] Other agents used include Afatinib, Brigatinib, Alectinib, Trametinib, Neratinib, Vandetanib, Pazopanib, Pertuzumab (1 each). [b] Other immunotherapy agents include-Pembrolizumab (2), Nivolumab (2), Ipilimumab (1), Sirolimus-1, Everolimus-1. [c] Following diagnosis of malignancy

Table S2. Correlation of different factors with intracranial disease-free survival and overall survival for the long-term survivors

Parameter	5-year intracranial disease-free survival	p-value	5-year Overall survival	p-value
**Age**<br>< 60 years<br>60 years or more	 37%<br>42%	 0.73	 69%<br>81%	 0.31
**Gender**<br>Male<br>Female	 38%<br>39%	 0.83	 52%<br>79%	 **0.04**
**ECOG**<br>0-1<br>2 or more	 41%<br>37%	 0.74	 76%<br>69%	 0.33
**No of metastasis**<br>Single<br>Multiple	 39%<br>37%	 0.33	 76%<br>65%	 0.30
**Site of metastasis**<br>Supratentorial<br>Infratentorial<br>Both	 31%<br>59%<br>36%	 <br>**0.04**	 70%<br>81%<br>64%	 <br>0.29
**Primary**<br>Lung<br>Breast<br>Melanoma	 32%<br>49%<br>60%	 <br>**0.06**	 75%<br>66%<br>100%	 <br>0.35
**Extracranial disease**<br>Controlled<br>Uncontrolled	 38%<br>39%	 0.91	 75%<br>70%	 0.62
**Diagnosis of BM**<br>Upfront<br>Diagnosed later (> 3 months)	 38%<br>39%	 0.53	 64%<br>77%	 0.78
**Systemic therapy**<br>Targeted/ HT/ Immunotherapy<br>None of above	 37%<br><br>41%	 0.64	 73%<br><br>71%	 0.71
**Timing of therapy**[a]<br>Before brain metastasis<br>During or after BM diagnosis	 49%<br>29%	 **0.13**	 69%<br>76%	 0.30
**Surgery during BM diagnosis**<br>Yes<br>No	 29%<br>44%	 0.47	 62%<br>79%	 0.85
**SRS during BM diagnosis**				

Yes	37%	0.80	71%	0.71
No	39%		74%	
**WBRT during BM diagnosis**				
Yes	39%	0.76	71%	0.90
No	38%		72%	

ECOG: Eastern cooperative oncology group; BM: Brain metastasis; HT: Hormonal therapy; SRS: Stereotactic radiosurgery; WBRT: Whole brain radiotherapy. [a] Targeted therapy, immunomodulators or hormonal therapy

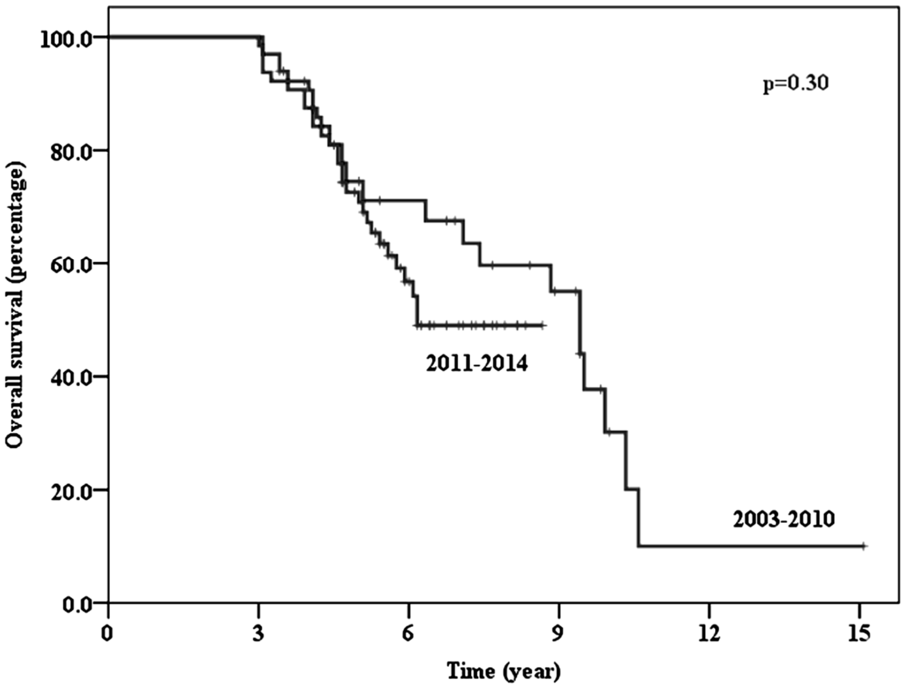

Numbers at risk

**2003-2010**	**33**	**33**	**20**	**11**	**1**	**1**
**2011-2014**	**64**	**64**	**23**	**0**	**0**	**0**

Figure S1. Kaplan Meier survival plot showing a comparison of overall survival for patients diagnosed with brain metastasis during 2003-2010 and 2011-2014.
